# Oral Health-Related Quality of Life After Allogeneic Bone Marrow Transplant—A Cross-Sectional Study †

**DOI:** 10.3390/healthcare13050561

**Published:** 2025-03-05

**Authors:** Nina Vovk, Manca Urek, Ksenija Cankar, Lidija Nemeth

**Affiliations:** 1 Department of Dental Diseases and Normal Dental Morphology, Faculty of Medicine, University of Ljubljana, 1000 Ljubljana, Slovenia; nina.vovk@mf.uni-lj.si; 2 Dental Center Osovnikar, 4220 Škofja Loka, Slovenia; manca.urek@gmail.com; 3 Institute of Physiology, Faculty of Medicine, University of Ljubljana, 1000 Ljubljana, Slovenia; ksenija.cankar@mf.uni-lj.si; 4 Division of Stomatology, University Medical Centre Ljubljana, 1000 Ljubljana, Slovenia

**Keywords:** dental caries, oral health, quality of life, xerostomia, bone marrow transplant, saliva, graft vs. host disease, haematology, cross-sectional study

## Abstract

**Objectives**: The aim of this study was to evaluate the oral health-related quality of life of patients with chronic graft-versus-host disease. **Methods**: A total of 22 patients with graft-versus-host disease aged 45.05 ± 14.66 years were enrolled in a single-centre cross-sectional study. Data from questionnaires on general health and diet, clinical examinations, and salivary tests were used to assess caries risks using the Cariogram computer programme. The Slovenian version of the Oral Health Impact Profile Questionnaire (OHIP-SVN) was used to determine the oral health-related quality of life. **Results:** Compared to healthy individuals, patients with chronic graft-versus-host disease had a lower oral health-related quality of life and a lower stimulated salivary flow rate (in both cases *p* < 0.001). The OHIP summary score correlated with stimulated salivary pH (R = 0.4916, *p* = 0.0277) and caries risk (R = 0.5420, *p* = 0.0111). **Conclusions**: In conclusion, our results confirm that cGVHD has a negative impact on oral health-related quality of life due to lower stimulated salivary pH and elevated caries risk (reduced salivary pH, flow rate, buffering capacity, and elevated *Streptococcus mutans* and *Lactobacillus* bacteria count). These findings emphasise the importance of a comprehensive assessment of oral health and preventive care in patients with cGVHD and suggest that the integration of clinical and quality of life measures could lead to improved patient care strategies.

## 1. Introduction

Chronic graft-versus-host disease (cGVHD) is a serious complication following allogeneic haematopoietic stem cell transplant that affects up to 80% of transplant recipients [1]. It can occur in children with an incidence of 20–40% and in adults with an incidence of around 50%, increasing to 60% with advancing age [2,3,4]. The disease manifests as a systemic multi-organ disease that typically affects the skin, eyes, mouth, liver, genitals, gastrointestinal tract, lungs, and musculoskeletal system [5]. The pathophysiology of cGVHD is complex and not fully understood. It involves donor autoreactive and alloreactive T and B cells that attack host tissues either by a direct response or by severe inflammatory reactions [6,7]. During treatment with multiple immunosuppressive drugs, the recipient’s immune system is no longer able to recognise its own cells, resulting in clinical features that resemble autoimmune diseases such as scleroderma or Sjögren’s syndrome [8,9,10].

Oral manifestations of cGVHD deserve special attention as the oral cavity is one of the most commonly affected sites and is often the primary site of disease manifestation. Oral cGVHD presents with various clinical features including mucosal changes, taste alterations, restricted tongue mobility, reduced mouth opening, desquamative gingivitis, and salivary gland dysfunction with xerostomia or mucocele [9]. The disease leads to the destruction of the salivary gland acini and thus to quantitative and qualitative changes in saliva, which are exacerbated by immunosuppressive therapy [11]. The impact on oral health is significant, as reduced salivary flow rate impairs the oral cavity’s immunity to infection and increases susceptibility to mechanical and chemical injury to the oral mucosa. Patients also have an increased risk of tooth decay, tooth loss, and the need for extensive dental treatment. In addition, these patients have a higher risk of developing oral squamous cell carcinoma [9,12]. The comprehensive care of cGVHD patients requires a multidisciplinary approach involving transplant specialists, dermatologists, ophthalmologists, dentists, and other medical professionals, depending on the organs involved. Long-term follow-up is essential to monitor the progression of the disease, manage complications, and adjust treatment strategies if necessary.

Dental caries is a chronic, multifactorial disease characterised by the demineralisation of hard dental tissues by bacterial acids [13]. The process begins with subclinical dissolution of the enamel, which can lead to visible non-cavitated and eventually cavitated lesions. Without treatment, caries can progress to pulp involvement, leading to inflammation (pulpitis) and, eventually, pulp necrosis [14,15]. Several key factors influence caries development. Saliva provides essential protective mechanisms through surface cleaning, acid buffering, bacterial control, and promotion of remineralisation. Reduced salivary flow rate significantly increases caries risk [16,17]. Dental plaque bacteria, especially *Streptococcus mutans* and *Lactobacillus*, create acidic conditions by lowering the pH of saliva below the critical value of 5.5 and promoting enamel demineralisation [18,19,20,21,22]. Recent in vitro studies have shown that *Streptococcus mutans* significantly lowers the salivary pH in the immediate vicinity of the tooth surface and creates a highly acidic microenvironment with pH values between 5.1 and 4.06 [23,24]. Dietary habits, especially the frequent consumption of fermentable carbohydrates, significantly impact caries development [25,26]. Regular fluoride supplementation through various applications increases the resistance of teeth to acid dissolution and promotes remineralisation, thereby reducing caries susceptibility by 30–70% [19,27,28].

Maintaining optimal oral health is essential, with a focus on preventing dental problems such as dental caries. In cases where carious lesions develop, early detection plays a crucial role in minimising further damage and maintaining overall oral health. The International Caries Detection and Assessment System (ICDAS) is an agreement on the clinical assessment of the caries process using the clinical visual–tactile method [29]. It is a classification of carious lesions according to their clinical appearance, which contributes to a better diagnosis, prognosis, and correct treatment. The lesions are classified into stages according to their histological extent in the tooth. The lesions are described with codes from 0 to 6, depending on their stage of development and whether they are active or inactive, depending on their character. Code 0 means a healthy tooth, codes 1 and 2 are non-cavitated lesions and codes 3–6 are cavitated lesions. Determining the activity of the lesion is an important factor in the choice of treatment, as active lesions require non-operative or operative treatment, while inactive lesions require improvement in oral hygiene and dietary habits as well as the use of fluoride [29,30].

Determining a patient’s likelihood of developing dental caries is essential for establishing an effective treatment plan. However, this assessment is challenging as caries development is caused by numerous interacting factors. The assessment needs to consider various personal characteristics that affect the balance between tooth mineral loss and its restoration [27]. The Cariogram software (Version 3.0j) provides an unbiased tool for caries risk assessment. This freely accessible programme calculates the likelihood of developing new caries by analysing several risk factors, including dietary habits (intake of sugary foods and drinks, and meal frequency), bacterial factors (dental plaque levels and presence of *Streptococcus mutans*), individual susceptibility (use of fluoride products, saliva buffering capacity and saliva production rate), and current health status (existing caries and related health conditions). [31].

The Oral Health Impact Profile (OHIP) questionnaire is one of the most widely accepted and comprehensive measures of subjective assessment of oral health-related quality of life, as its questions cover a wide range of areas that influence quality of life [32,33,34,35,36]. It assesses quality of life from three perspectives: physical, psychological, and social [37]. It is one of the most useful tools in oral health research to compare the success of different treatment methods and evaluate the effects of both oral diseases and their treatment on patient’s well-being [33,34,38,39,40,41]. OHIP-49 has been translated into Slovenian (OHIP-SVN) and is a suitable instrument to assess oral health-related quality of life in Slovenia [32]. OHIP-14, a shorter version of OHIP-49, is one of the most commonly used questionnaires to assess general and oral health-related quality of life in HSCT patients [42]. Analysing the results reported by patients has influenced treatment decisions and redefined therapeutic approaches. The introduction of these assessment tools is essential for improving patient care and treatment efficiency.

Research on oral cGVHD and its impact on oral health-related quality of life is still relatively scarce [43,44,45,46]. Existing studies focus primarily on mucosal changes, relationships between different cGVHD components and the development of treatment options to alleviate oral cGVHD symptoms [47,48]. However, there are few studies addressing caries and its prevention in these patients. While complex therapeutic procedures often dominate clinical attention, dental caries remains an important problem. Dental caries can cause significant pain, potentially leading to tooth loss, impedes food intake and significantly affects quality of life. The occurrence of new caries lesions following grafting procedures has been documented in the literature [49]. This is aggravated by cGVHD-related symptoms such as dry mouth, mucosal damage, and increased susceptibility to infection, all of which increase the risk of caries development. To improve the oral health of this vulnerable patient group, a structured interdisciplinary approach involving the patient, the dentist and the haematologist is essential. Preventive measures should be taken prior to bone marrow transplant and patient behaviour should be managed to minimise the negative consequences of treatment and cGVHD.

The main motivation for this study stems from the growing awareness among patients and healthcare professionals of the significant impact of cGVHD on oral health. Therefore, the aim of this study was to determine the impact of cGVHD on oral health-related quality of life. Our hypothesis was that cGVHD has a negative impact on oral health-related quality of life.

## 2. Materials and Methods

The present study is a single-centre, cross-sectional observational study that follows the reporting guidelines outlined in the STROBE Statement [50].

**Figure 1:**
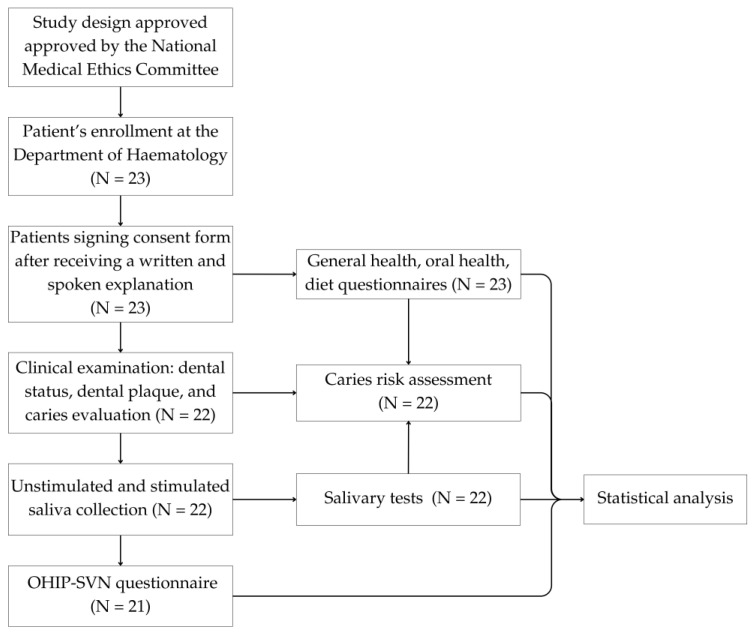
The flowchart of patient recruitment and technique.

A total of 22 adult patients who had undergone allogeneic haematopoietic stem cell transplant and had been diagnosed with cGVHD were included in this study. The inclusion criteria were a diagnosis of cGVHD and the presence of at least 3 teeth per quadrant (one from each group of teeth). The exclusion criteria were the presence of less than 3 teeth per quadrant and the use of a removable denture. The flowchart of patient recruitment and technique is shown in Figure 1.

All patients were invited during their regular clinical visit at the Department of Haematology of the University Clinical Centre Ljubljana and informed about the aim and procedure of the study and received a written explanation. Patients voluntarily and consciously participated in the study. Before the clinical procedures, all patients signed a consent form for the present study. It was approved by the National Medical Ethics Committee of the Republic of Slovenia under the number 0120-400/2019/10 on 20 August 2019.

Patients first completed a questionnaire on their general health, oral health, and diet. Clinical examinations were performed by the same calibrated examiner who had completed ICDAS baseline and clinical training under the supervision of two experts who are senior members of the ORCA (Organisation for Caries Research) [51,52,53]. All tooth surfaces were assessed by the visual–tactile method using the ICDAS criteria [29]. All clinical examinations were performed in a uniform environment with uniform lighting conditions and facilitated by air-drying. Caries risk factors evaluated were unstimulated and stimulated salivary flow rate, pH of stimulated and unstimulated saliva, buffering capacity of stimulated saliva, and colony density of *Lactobacillus* and *Streptococcus mutans* bacteria. The Cariogram computer programme (Version 3.0j) was used to assess caries risk using data from questionnaires on general health, oral health and diet, clinical examination, and salivary test results [31].

The Oral Health Impact Profile—Slovenian version (OHIP-SVN), which consists of 49 questions, was used to assess the participants’ oral health-related quality of life [32]. Participants completed the questionnaire following their clinical examination. The questionnaire assesses categories such as limited functionality, psychological difficulties, physical pain, and physical, psychological, social, and general impairment. The questionnaire also assesses the participant’s perception of their oral health and appearance. The questionnaire asks participants how often they have had certain problems in recent months. The answers are analysed on a Likert scale: 0 means never, 1 rarely, 2 occasionally, 3 often, and 4 always. Zero means that a particular problem is not present, while the highest value indicates a clearly present problem [54]. The values of the individual categories were then added together to obtain the OHIP summary score.

The independent variables were caries risk factors: unstimulated and stimulated salivary flow rate, pH of stimulated and unstimulated saliva, buffering capacity of stimulated saliva, colony density of *Lactobacillus* and *Streptococcus mutans* bacteria, and caries risk (independent variables). The dependent variables were scores from the domains of the OHIP-SVN questionnaire: limited functionality, psychological difficulties, physical pain, physical, psychological, social and general impairment, and the OHIP summary score. Statistical analysis was performed using SigmaPlot 14.0 (Systat Software, San Jose, CA, USA). The sample size was determined based on a power of 0.8, a correlation coefficient of 0.5 and a significance level (α) of 0.05. The estimated sample size required was N = 30. The descriptive statistics are given as arithmetic means (M) with standard deviations (SD), medians and interquartile ranges (25th and 75th percentiles). The criterion for determining statistical significance was a *p*-value of less than 0.05. The Shapiro–Wilk test was used to assess normality. To assess the correlation between the measured parameters (unstimulated and stimulated salivary flow rate, pH of stimulated and unstimulated saliva, buffering capacity of stimulated saliva and colony density of *Lactobacillus* and *Streptococcus mutans* bacteria, OHIP-SVN questionnaire results, and caries risk), linear regression analysis was applied, using Pearson’s correlation for parametric variables and Spearman’s correlation for non-parametric variables. Student’s *t*-test was used to compare the means and standard deviations of the OHIP sum score between our group and the values of the normal population (n = 400) reported in a study by Rener et al. [55]. The mean values of the unstimulated and stimulated salivary flow rates of our study group were compared with healthy control values (n = 441) from a study by Bergdahl et al. using the Wilcoxon test [56].

## 3. Results

Salivary samples were taken from a total of 21 patients. One patient withdrew from the study because his blood profile showed signs of deterioration. The second patient did not have enough saliva, so we were unable to collect a sample. The unstimulated and stimulated salivary flow rate values of the latter patient were counted as 0 mL/min. Salivary tests were performed; however, in one of the remaining 21 patients, the amount of saliva was not sufficient to perform a bacterial analysis (colony density of *Streptococcus mutans* and *Lactobacillus* bacteria). The OHIP questionnaire was completed by 21 patients.

### 3.1. Results of the Clinical and Demographic Data and the OHIP-SVN Questionnaire

The results are shown in Table 1 and Table 2.

### 3.2. The Relationship Between the Measured Parameters and the Results of the OHIP-SVN Questionnaire

**Figure 2:**
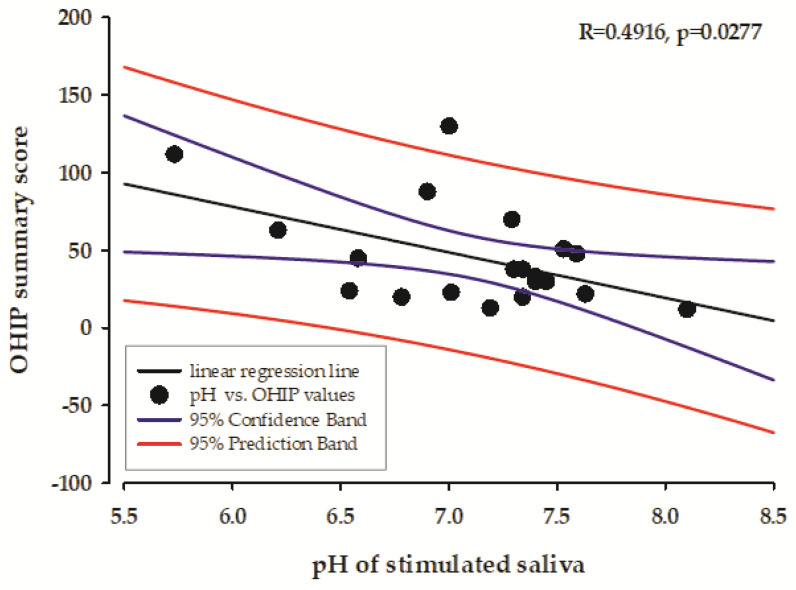
The correlation between the pH value of stimulated saliva and the OHIP summary score.

**Figure 3:**
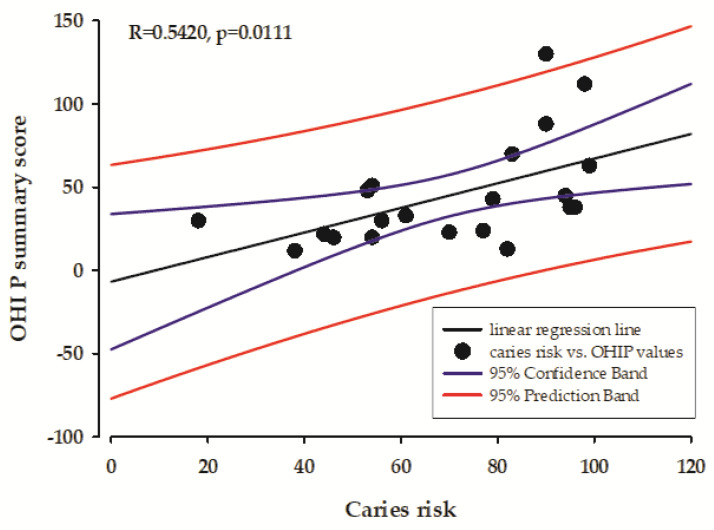
The correlation between caries risk and the OHIP summary score.

Regarding the caries risk factors the pH value of the stimulated saliva and caries risk were correlated with the OHIP summary score (Figure 2 and Figure 3).

The unstimulated salivary flow rate correlated with psychological discomfort and social disability. Correlations were also found between the stimulated salivary flow rate and functional limitation, physical pain, psychological discomfort, physical disability, handicap, and self-reported oral health. The pH value of the stimulated saliva was correlated with social disability. The correlation coefficients (R) and statistical significance (*p*-value) are shown in Table 3.

Deep caries lesions (ICDAS 5 and 6) correlated significantly with the OHIP summary score (R = 0.592, *p* = 0.00471).

### 3.3. Differences Between cGVHD Patients and the Healthy Population

The OHIP summary score was significantly higher in the group of cGVHD patients compared to the healthy population (*p* < 0.001). The unstimulated salivary flow rate was lower in the cGVHD group than in the healthy population, but the difference was not significant (*p* > 0.05). The stimulated salivary flow rate was significantly lower in the cGVHD group compared to the healthy population (*p* < 0.001).

## 4. Discussion

### 4.1. General Findings

The aim of this cross-sectional study was to investigate oral health-related quality of life in patients with cGVHD. It is known that cGVHD patients suffer from various manifestations in the oral cavity. In our study, we focused on the investigation of risk factors for the development of dental caries.

According to the results of a study by Rener-Sitar et al. with 400 healthy participants, our patients reported poorer oral health-related quality of life, and the difference was statistically significant [55]. This was to be expected, as we found reduced salivary flow in our samples, which had a negative impact on oral health and thus on the quality of life. Consistent with the results of our study, Stolze et al. reported that the quality of life related to oral health as measured by the OHIP-14 was impaired and mainly negatively affected by complaints of oral pain and oral sensitivity rather than by the severity of oral mucosal cGVHD [43].

Compared to healthy controls reported by Bergdahl et al., both unstimulated and stimulated salivary flow rates were lower in patients with cGVHD [56]. The decrease in stimulated salivary flow rate reached statistical significance, while the decrease in unstimulated flow rate was not significant. This result could be attributed to the greater impairment of the parotid glands in cGVHD, which are primarily responsible for stimulated saliva production [57]. Daikeler et al. showed in their larger study of 44 cGVHD patients and 44 healthy controls that both stimulated and unstimulated salivary flow rates were significantly reduced in cGVHD patients [58].

The results of our study showed a moderately negative correlation between the total OHIP value and the pH value of the stimulated saliva. This result suggests that individuals who have more oral health problems and poorer quality of life (indicated by higher OHIP scores) tend to have more acidic stimulated saliva. This correlation can be explained by the complex interplay between oral health status and salivary parameters. Impaired salivary gland function and altered saliva composition can lead to a reduced buffering capacity, resulting in a more acidic oral environment, which in turn leads to a deterioration in oral health. Conversely, this altered pH can further compromise oral health, creating a cyclical relationship between salivary dysfunction and deterioration of oral health. This finding contributes to our understanding of the relationship between subjective oral health measures and objective physiological parameters and points out the importance of considering both in clinical assessment and treatment planning. In addition, our results showed a moderately positive correlation between overall OHIP scores and caries risk, which is consistent with our current understanding of oral health dynamics. This correlation suggests that individuals with higher OHIP scores, reflecting poorer oral health-related quality of life, also have a higher caries risk. This correlation is logical, as the factors that contribute to poor oral health-related quality of life often overlap with the factors that increase caries susceptibility, such as poor oral hygiene, dietary habits, and impaired salivary function. This finding highlights the importance of considering patient-reported outcomes alongside clinical risk assessment in the comprehensive assessment of oral health.

To date, studies that have investigated this relationship in the adult population are relatively rare. There are studies in the literature that find correlations between other important variables and self-rated general health. In the study by Rødseth et al., the results showed an association between caries experience and self-rated general health [59]. Silva et al. investigated the possible associations between the clinical consequences of untreated caries and self-rated oral health in 12-year-old adolescents; however, no statistically significant associations were found [60].

The moderately positive correlation between deep carious lesions (ICDAS 5 and 6) and the total OHIP score observed in our study emphasises the significant impact of severe caries on the patient’s quality of life. Similar correlations between severe caries and impaired quality of life have also been found in other populations, suggesting that early caries treatment may be critical to maintaining a better quality of life in these patients. In a study conducted by Leal et al., a poorer quality of life was observed in children with a greater number of tooth surfaces with cavitated dentin lesions [61]. These lesions caused pain and made it difficult to chew with the affected teeth due to sensitivity. Alanzi et al. demonstrated that both the presence and extent of untreated early childhood caries significantly impair oral health-related quality of life in preschool children and their families [62].

Psychological issues among our participants, such as worry about teeth, discomfort, feelings of misfortune, and tension related to oral health problems, were statistically significantly associated with salivary parameters that contribute to caries formation and progression as well as poorer dental status. Additionally, we found that a poorer self-reported oral health status was associated with higher caries risk. A higher caries risk was also associated with poorer quality of life, as shown by the results of the OHIP questionnaire.

CGVHD affects the quality of life of patients in many ways and thus differs significantly from the general healthy population [1,63]. These comprehensive effects go beyond physical symptoms and affect psychological well-being, social interactions, and daily activities. Our findings are consistent with previous studies that have documented the significant burden of cGVHD on patients’ overall functioning and well-being [43,44,45,46]. The multiple manifestations of cGVHD, including oral symptoms, can lead to significant limitations in daily activities such as eating, speaking, and social interactions that are often taken for granted in healthy individuals. In addition, the chronic nature of these symptoms can lead to ongoing psychological distress and social isolation, creating a cycle that further limits the quality of life. This highlights the importance of a holistic approach to patient care that considers not only the physical manifestations of cGVHD, but also its psychological and social impact [64]. Understanding these differences from the general population is critical for healthcare providers to develop targeted interventions and support strategies that can improve the overall well-being of patients with cGVHD.

Saliva plays a vital role in maintaining oral health through its multiple protective functions. It helps with lubrication, buffering, remineralisation, antimicrobial activity, and facilitates taste perception and digestion. A reduced salivary flow rate (hyposalivation) has a significant impact on oral health and quality of life. Patients with hyposalivation often experience xerostomia (dry mouth sensation), have difficulty speaking, eating, and swallowing, and need to drink water frequently. The condition can lead to various oral manifestations, including loss of mucosal glossiness, development of thin and cracked oral mucosa, tongue fissures, angular cheilitis, and increased susceptibility to oral infections, particularly candidiasis [65]. A study by Santos-Silva et al. described the clinical features of aggressive dental caries in patients with cGVHD [66]. The pattern of caries distribution observed in patients with head and neck tumours after radiotherapy is similar to that of patients with salivary gland impairment and consequent hyposalivation, in whom caries develops in typically resistant areas, including cervical regions, incisal edges of the anterior mandibular region, smooth surfaces and root surfaces [66].

Psychological recovery after bone marrow transplant is typically longer than physical recovery, particularly in patients with persistent cGVHD [67,68]. While most transplant survivors demonstrate high resilience, they face numerous emotional challenges including uncertainty, fear of relapse, anxiety, and depression. Factors associated with increased vulnerability to emotional difficulties include traumatic transplant experience, lower levels of education, lower income, and poorer pre-transplant mental health [69,70]. Notably, persistent cGVHD and poor current health status correlate with more severe psychological symptoms. Despite these challenges, less than half of survivors with psychological needs receive appropriate treatment [71].

In our study, no other statistically significant differences were found between the severity of cGVHD and different underlying diagnoses, in relation to the quality of life associated with oral health. However, these results should be interpreted with caution as our sample was relatively small and therefore we may only have been able to detect small differences. Further research with larger cohorts would be valuable to confirm these observations and explore additional factors that may influence oral health-related quality of life in this patient group.

The overall assessment of caries risk includes salivary parameters, participants’ previous experiences with carious lesions or dental status, plaque on tooth surfaces, diet, fluoride intake, and systemic diseases that directly or indirectly affect the oral health of patients with cGVHD [31]. A high caries risk means that individuals have a low chance of avoiding new carious lesions in the future, which would lead to a further deterioration in their oral health and quality of life. This situation could be improved by preventive dental visits at the time of initial disease diagnosis and prior to bone marrow transplant. By incorporating dental care into the initial treatment planning, healthcare providers could take a more proactive approach to maintaining patients’ oral health, which could reduce complications and improve overall quality of life throughout the course of the disease. This is especially important as it is the only way to break the vicious cycle created by the dynamics described above.

### 4.2. Strengths, Limitations, and Recommendations

The greatest strength of our study is its clinical applicability and its focus on prevention. The results emphasise the importance of early identification of patients at risk of compromised oral health-related quality of life so that timely intervention is possible. This approach is particularly valuable as it allows dentists to take caries prevention measures before the onset of cGVHD, when patient compliance is likely to be higher. An early understanding of the impact of cGVHD on quality of life may facilitate better patient engagement in prevention protocols and potentially lead to better long-term oral health outcomes.

A major limitation of this study was that it coincided with the COVID-19 pandemic. The resulting healthcare restrictions led to fewer bone marrow transplants and lower patient compliance. Although we reached our originally estimated sample size, pandemic-related circumstances forced an early cancellation of the study, resulting in attrition of participants and some missing data points. These limitations should be considered when interpreting our results. To address these limitations, we are planning a larger longitudinal study in which patients will be followed from pre-transplant baseline to the post-transplant period. The study will monitor caries risk factors and systematically assess changes in oral health parameters over time. This comprehensive approach will not only lead to a better understanding of caries development patterns but will also support the development of targeted prevention strategies. Based on the identified risk factors, individualised causal treatment protocols will be developed to minimise caries development and maintain optimal oral health in this vulnerable patient group.

This study aimed to raise awareness among patients, dentists, and haematologists of the problems related to compromised oral health that can be caused by cGVHD. The implementation of preventive measures prior to transplant is crucial for the causal treatment of these patients. Essential preventive strategies include education and motivation to maintain excellent oral hygiene, dietary changes to reduce frequent consumption of simple carbohydrates, the use of fluorides, and regular dental visits. These preventive measures are in line with current recommendations for the treatment of patients at high risk of caries. During treatment, it is extremely important to examine patients regularly and treat any problems in the oral cavity. After transplant and cGVHD diagnosis, maintaining optimal oral health through comprehensive care is crucial for these patients.

## 5. Conclusions

Our study hypothesis that cGVHD affects the oral health-related quality of life was confirmed by our results. The obtained data provide valuable insights into the oral health-related quality of life of patients with cGVHD. The observed correlations between OHIP-SVN scores, stimulated salivary pH, and caries risk demonstrate the complex interplay between objective oral health parameters and the subjective experience of patients. These findings point out the critical role of dental professionals throughout the patient’s course of treatment, starting with preventive measures even before bone marrow transplant. It is essential to increase patients’ awareness and understanding of oral health. By demonstrating these links between oral health parameters and quality of life, we aim to positively influence patients’ behaviour and improve their engagement in preventive oral care, which is crucial for caries prevention in this high-risk group.

## Figures and Tables

**Table 1:** Clinical and demographic characteristics of the sample (N = 22).

	Frequency (N)	%	Mean (SD)	Median (25%, 75% Percentiles)
Age	22		45.05 (14.66)	47.5 (25% 29.75, 75% 58.25)
Gender				
Male	10	45.45		
Female	12	54.55		
BMI	22		22.86 (4.13)	22.5 (25% 20.05, 75% 25.63)
Race				
Caucasian	22			
Smoking				
Yes	0	0		
No	22	100		
Diagnosis				
Acute myeloid leukaemia	14	63.64		
Acute lymphoblastic leukaemia	6	27.27		
Mielodysplastic syndrome	1	4.55		
Non-Hodkin lymphoma	1	4.55		
Time since transplant (months)	22		27.55 (48.87)	
NIH cGVHD Global Rating				
Mild	15	68.18		
Moderate	5	22.73		
Severe	2	0.09		

**Table 2:** Results of the OHIP-SVN questionnaire (N = 21).

Domain Score	Mean (SD)	Median (25%, 75% Percentiles)
Self-reported oral health	2.21 (0.9)	3 (25% 2, 75% 3)
Self-reported oral aesthetic	2.09 (0.86)	2 (25% 2, 75% 3)
Functional limitation	10.50 (7)	9 (25% 6, 75% 15)
Physical pain	6.95 (4.69)	5 (25% 4.5, 75% 11.5)
Psychological discomfort	7.09 (5.33)	5 (25% 4, 75% 10.5)
Physical disability	7.01 (6.93)	5 (25% 0.5, 75% 11.5)
Psychological disability	3.23 (4)	2 (25% 0.5, 75% 4.5)
Social disability	1.32 (2.17)	0 (25% 0, 75% 1.5)
Handicap	2.91 (4.15)	1 (25% 0, 75% 4)
OHIP summary score	40.81 (31.83)	23 (25% 8, 75% 46.5)

**Table 3:** Correlations between the measured salivary parameters and the results of the OHIP-SVN questionnaire (R (*p*-values)).

N = 21	pH of Unstimulated Saliva (N = 21)	Unstimulated Salivary Flow Rate (N = 21)	pH of Stimulated Saliva (N = 21)	Stimulated Salivary Flow Rate (N = 21)	Caries Risk (N = 21)
Self-reported oral health	NS	NS	NS	NS	−0.512 (0.0177)
Self-reported oral aesthetic	NS	NS	NS	NS	NS
Functional limitation	NS	NS	NS	NS	0.499 (0.0214)
Physical pain	NS	NS	−0.516 (0.0198)	NS	0.439 (0.0466)
Psychological discomfort	NS	−0.442 (0.0447)	−0.495 (0.0266)	NS	0.622 (0.0026)
Physical disability	NS	NS	NS	NS	0.469 (0.0322)
Psychological disability	NS	NS	−0.453 (0.0448)	NS	0.475 (0.0296)
Social disability	NS	NS	NS	NS	NS
Handicap	NS	NS	NS	NS	NS

NS: non-significant.

## Data Availability

The complete data will be sent on request.
